# Dietary Practices and Barriers to Adherence to Healthy Eating among King Faisal University Students

**DOI:** 10.3390/ijerph17238945

**Published:** 2020-12-01

**Authors:** Amal Ismael Abdelhafez, Fahima Akhter, Abdulrahman Abdulhadi Alsultan, Sahbanathul Missiriya Jalal, Ayub Ali

**Affiliations:** 1 Department of Nursing, College of Applied Medical Sciences, King Faisal University, Al-Ahsa 31982, Saudi Arabia; falamin@kfu.edu.sa (F.A.); sjalal@kfu.edu.sa (S.M.J.); 2 College of Applied Medical Sciences, King Faisal University, Al-Ahsa 31982, Saudi Arabia; aalsultan@kfu.edu.sa; 3 Department of Family & Community Medicine, College of Medicine, King Faisal University, Al-Ahsa 31982, Saudi Arabia; drayub@kfu.edu.sa

**Keywords:** dietary practice, healthy diet, adherence, university students, barriers, eating habits

## Abstract

Proper dietary practices should be developed during the student years that will continue into the future. This study aimed to identify the eating habits and dietary practices among King Faisal University (KFU) students, explore the barriers to adherence to healthy eating, associate the understanding of healthy diets with students’ characteristics, and determine the association between body mass index (BMI) and awareness of the concept of healthy diets, academic discipline, and enrollment in a nutrition course. In this cross-sectional study, students were selected randomly and a questionnaire was distributed using an electronic platform through KFU email. Out of 564 students, nearly half (45.7%) reported eating snacks as their main food, and some (38.3%) reported eating with their family twice daily. The students rarely reported eating with friends (73%) or eating dates (48.8%). Furthermore, many reported that they were not consuming a balanced diet (42.6%). Some students (46.3%) reported taking breakfast daily, and 49.1% reported eating meals regularly. There was low consumption of vegetables (29.3%) and fruits (26.2%) among the students. The barriers to adherence to healthy eating were the availability of fast food (73.2%), high cost of healthy food (72.7%), limited time (59%), and laziness (57.1%). Statistically significant data indicated that the students with a normal BMI were more aware of the concept of healthy diets, studied medical and applied sciences, and were enrolled in KFU nutrition courses.

## 1. Introduction

The dietary practices of university students will carry over into later life, playing a long-term role in dietary behavior and food consumption, as this is an essential period in habit formation [1,2]. Students are the future of the nation, and are expected to have sufficient knowledge about dietary patterns and appropriate dietary practices. In 2012, the Ministry of Health, Kingdom of Saudi Arabia, published food-based dietary guidelines (FBDG) that promote a healthy diet and aim to change living styles in the wider population [3]. During the last three decades, dramatic changes have been observed in socio-demographic and individual lifestyles in Saudi Arabia due to increased urbanization and rapid economic growth, which have impacted on the patterns of food consumption and led to increasingly unhealthy diets [4].

The important factors that influence the patterns of dietary consumption are eating habits, changes in perceived knowledge about nutrition, income status, and cultural beliefs, as well as social, economic, and geographical limitations. Diets with a high energy value, with added fat and sugars and reduced intake of fiber-containing foods and complex carbohydrates, are the leading cause of non-communicable diseases such as heart problems, stroke, kidney diseases, diabetes mellitus, hypertension, osteoporosis, and cancer [5,6,7]. Furthermore, students are more likely to drink coffee, tea, and soda than fruit juice or plain water to make up the fluid balance of their body. High coffee consumption is associated with stroke, coronary heart disease, heart failure, liver cirrhosis, urinary tract infection, stomach upset, and more [8]. On the other hand, fruit juices are a rich source of vitamin C, carotenoids, and polyphenols, with health-promoting benefits [9].

University students can struggle with many barriers that may inhibit them from adhering to healthy diet practices in their daily life. These barriers can be due to poor physical activity, ignorance, and environmental factors [10,11]. In addition, many factors such as a lack of skill in choosing healthy diets and negative coping strategies for stress during study periods can negatively affect eating behaviors. Further, students often report not being interested in preparing healthy foods and undertake only periodical assessment of their nutritional status [1,12,13]. Moreover, the variety of food availability influences students’ preferences in their selection of food. The most documented factors related to student diets are difficultly cooking healthy food, ease of junk food preparation, limited availability of healthy food items, palatable taste, attractive advertising of unhealthy diets, and the high cost of healthy foods [14].

For these reasons, it is a challenge to promote qualitative changes in food consumption and adaptation to healthy dietary practices among university students. Providing nutritional programs at the university level to create awareness among the students of nutritional value and balanced diets has been recommended as an effective way to prevent some non-communicable diseases [15,16]. Though various studies have assessed the nutritional status of Saudi citizens, there has not been a study performed with the students at King Faisal University prior to this. This study aimed to identify the eating habits distribution and dietary practice of the students, explore the barriers to adherence to healthy eating, associate the understanding of healthy diets with students’ characteristics, and determine the association between body mass index (BMI) and awareness of the concept of healthy diets, academic discipline, and enrollment in a nutrition course.

## 2. Material and Methods

### 2.1. Settings and Population

This study was conducted at King Faisal University (KFU), Al-Ahsa, which is in the eastern province region of Saudi Arabia. KFU is the country’s public university and has 21,870 students in different under-graduate programs, with female students comprising 68% of the student population, and male students 32%. The respondents were students studying in various disciplines in the university, either medical and applied sciences or humanities, arts, and social sciences. The health promotion courses were offered for all disciplines.

### 2.2. Study Design and Sampling

This was a cross-sectional study with a descriptive method. Open source epidemiologic statistics from the public health website www.OpenEpi.com were used to calculate the sample size. From the total population of 21,870 students, with a 95% confidence and 80% power, the total sample size was calculated to be 642. Based on the limitations and expected deficiencies in the online responses, 50% was added and a total final sample size of 642 was the aim. However, by the closing date, a total of 564 responses were received and analyzed. The enrolled students from various disciplines were randomly picked by computerized generated randomization allocation, with the assistance of software.

### 2.3. Data Collection Tool and Procedure

The data were collected using a structured questionnaire from 1 September to 30 September 2020. The tool was developed after reviewing the literature [11,15,17,18,19,20,21], and was evaluated by experts.

The questionnaire consisted of three parts. Part one queried the characteristics of the students, including age in years, gender, academic level, academic disciplines, marital status, family type, BMI, participation in nutritional education, and awareness of the concept of healthy diet. The second part covered the students eating habits and dietary practices. The third part of the questionnaire included questions about barriers to adherence to a healthy diet.

The questionnaire was developed in English and translated into Arabic. The Arabic version was translated back to English to verify the consistency of the translation. The questionnaire was piloted among 10 students, and they were excluded from the final analysis. The reliability of the questionnaire was tested (*r* = 0.987) using Cronbach’s alpha. The time to fill in the questionnaire ranged from 10 to 15 min.

An online version of the structured questionnaire was developed via Google forms, and the link was sent to the selected students at various colleges at KFU. The survey tool was made available for 30 days to allow the students to respond at a convenient time, but at one attempt. An introduction was added explaining the objectives of the study and ensuring privacy and confidentiality before distribution. Participation in the study was voluntary, and informed consent was obtained electronically before the survey.

### 2.4. Ethical Considerations

Ethical approval was obtained from the Research Ethics Committee, Deanship of Scientific Research, KFU (HAPO-05-HS-003). All students were asked to agree before participation, and given information about confidentiality, the lack of risk, anonymity, and voluntary participation.

### 2.5. Statistical Analysis

Statistical Package for Social Sciences (SPSS), version 21.0, Armonk, NY: IBM Corp was used to analyze the study data. The numbers and percentages were tabulated in the form of frequency distribution by using descriptive analysis. Chi-square analysis was used to test the association between students’ awareness of the concept of healthy diet and the characteristics of the students. The associations between BMI and academic discipline and enrollment in a nutrition course were analyzed. Statistical significance was fixed at *p* < 0.05.

## 3. Results

Table 1 displays the characteristics of the students in the study. The total number of participants was 564, and the mean age score was 20.45 ± 1.64 years. Regarding academic levels, 67.6% were registered in preparatory to second year, and 32.4% were enrolled in third year to the internship year. In terms of academic disciplines, 76.2% of the students were in medical and applied sciences, whereas 23.8% were in humanities, arts, and social sciences. Most of the students (86.5%) were female. The results also show that 56.9% of the students belonged to a simple family, and 53.9% of the students had a normal BMI. Among them, 71.6% were enrolled in a nutrition course, and 84.9% were aware of the concept of a healthy diet.

Table 2 shows that 45.7% of the students reported having snacks twice daily, and 25.7% and 20.7% ate vegetables and fruit once per day, respectively. A total of 48.2% reported rarely eating dates. Additionally, 36.3% and 37.8% reported eating fried food and fast food 1–2 times per week, respectively. Moreover, above sixty percent (61.3%) reported eating with family either once (23%) or twice (38.3%) per day. The findings showed that 73% of the students reported rarely eating with friends, which was rare. Furthermore, 42.6% of the students reported that they rarely consumed a balanced diet.

Table 3 illustrates the frequency of dietary practices among the participants. Half of the students (50%) reported that they always add iodized salt while cooking food. Near to half (49.1%) ate meals regularly, and 46.3% ate breakfast daily. When choosing food, 40.8%, 31.2%, and 36.5% of the students sometimes considered plant sources, considered the nutrient value, and followed a healthy diet, respectively.

Table 4 describes the barriers to adherence to eating a healthy diet among the students. The highest agreed barriers were food related factors, in that 73.2% of the students specified that fast foods were easily and quickly available, and 72.7% mentioned that healthy foods were costly. The other barriers were limited time to prepare healthy food (59%) and being too lazy to prepare healthy food (57.1%). On the other hand, around half of the students (50.4%) disagreed that pressure from peers to engage in unhealthy eating acting was a barrier. Moreover, there were statistically significant (*p* ≤ 0.05) differences between all barriers in terms of their impact on adherence.

Table 5 shows no association between awareness of the concept of healthy diets and age, family type, gender, or academic discipline. On the other hand, a statistically significant association was found between awareness of the concept of a healthy diet and marital status (*p* = 0.001), academic level (*p* = 0.031), and attending nutrition courses provided at the university (*p* = 0.0001).

Table 6 shows the statistically significant association between BMI and awareness of the concept of a healthy diet (*p* = 0.04), academic discipline (*p* = 0.001), and nutrition course enrollment (*p* = 0.049).

## 4. Discussion

In the present study, the eating habits of students were found to be inadequate to some extent. This is in line with the findings of many studies indicating that college students’ dietary practices tend to be unhealthy [15,16,22,23].

In our study, around half of the students reported eating breakfast and meals regularly. This can be attributed to the clinical and practical hours in most colleges beginning in the early morning; many students live far from the university and may not have time for breakfast. Moreover, reluctance to eat a regular breakfast can be due to studying-associated stress and exams. This could also be the reason that near to half of the studied students were eating snacks as their main food. This is in line with a study done in Abaha and Jeddah, which reported that 49% and 15% of their subjects skipped a regular breakfast, respectively [8]. Additionally, in most cases, students preferred to take snacks as their breakfast and lunch, and preferred to buy these snacks from restaurants or the university cafeteria [20]. A similar study evidenced that most students (83.6%) regularly eat three meals per day and understand the concept of a balanced diet, but only 7% follow this for their own diet, yet 51% showed a tendency towards learning more about healthy diets [24].

The current findings showed that most students did not eat with friends, and tended to eat with their family once or twice daily. This can be attributed to the majority being female, and living in simple or joint families. This is not in line with other studies which found that most students avoid eating homemade food because they prefer to eat with their friends outside the home. In addition, they ignore the nutritional value labeled on the food items. When they choose a meal in a restaurant, they prefer to eat delicious and diverse food, and snacks with high fat and calorie values [9,12].

Our study showed that there was a low consumption of dates, vegetables, and fruits, and overall a low rate of a balanced diet among the students. This is similar to research which showed that college students are often unable to meet the required intake of fruits and vegetables, frequently intake of snacks on foods high in fat and calories, tend to skip breakfast, and have a higher rate of fast food consumption [17]. Moreover, this is in line with the findings of a similar Australian study [25].

Regarding the BMI status of the students, in the current study, among all participants, 53.9% had a normal body weight, while 22.8% were overweight or obese. Similarly, a study done on 357 Saudi students enrolled in Al Qassim University, KSA, in 2009 reported that 57.3% of the students were within the normal body weight range, whereas 37.5% were overweight or obese [26].

Food is essential for the health of human beings as it delivers energy and nutrients [27]. Every student should have access to information about nutritional requirements. School-aged students depend on their parents and follow their advice in their selection of foods. However, the transition from school to college life gives more freedom in the type and the amount of food consumption. Some studies related to awareness of a healthy diet have proven that students have a fair knowledge of nutritional requirements for health [18,28,29,30,31]. In our study, 71.6% of the students were enrolled in a nutrition course, and 84.9% understood the concept of a healthy diet.

Regarding dietary practice frequency among the college students, our study showed that half of the students always add iodized salt while cooking food. Less than half of the students consider plant sources, nutrient value, or follow a healthy diet when they choose food. These results agree with the findings of several studies [32,33,34,35].

As indicated by some researchers [1,2,36,37], the high cost of some food stuffs, accessibility and availability of food, and lack of motivation in food preparation were the strongest barriers to a healthy diet. Hence, professionals and responsible authorities from nutrition organizations need to design programs and develop tools that can help students to be more motivated in choosing healthy foods at the university level. The college students realized, during focus groups, that their eating habits and college facilities play a strong role in creating barriers. In the preparatory period, students start college and need to adapt to a new environment, and new dining habits with new friends. These factors also have an impact on their food selection and eating habits, and their tendency to maintain healthy behaviors [16,38,39]. However, our study found that there are some other barriers to adherence to eating a healthy diet among students. The highest agreed barriers to eating a healthy diet we found were the easy availability of fast foods, high cost of healthy foods, limited time, and being too lazy to prepare healthy meals. On the other hand, the lowest agreed barrier was pressure from peers to engage in unhealthy eating.

This study proved that there are statistically significant relationships between understanding of the concept of a healthy diet and marital status, BMI, and education about food and nutrition, indicating that the students who understand the meaning of a healthy diet tend to be single, with a normal BMI, and have received nutritional education. Studies have shown that most students are not acquainted with necessary healthy foods of different categories and are in need of educational programs about healthy food and nutrition [19,24,40]. This is in line with a cross-sectional study conducted at Kuwait University to assess the body mass index, eating habits, attitudes, calculation of healthy eating scores, and gender differences among these domains. A total of 615 students participated in this study. The results show that most of the men were overweight and obese, compared with the women. Both men and women had equally unhealthy dietary habits [41]. On the contrary, no correlation was found between knowledge regarding nutrition and BMI, indicating that knowledge alone does not necessarily promote an adequate BMI [28].

With regards to nutrition status, this paper found a statistically significant association between BMI and academic discipline and nutrition course enrollment, indicating that students who had normal BMI were more likely to be in the medical and applied colleges, and attend a nutrition course. This can be attributed to the effect of knowledge on changing dietary practices, with the outcome of a normal BMI. This finding is similar to a previous study [18] which reported a relationship between normal BMI and nutrition academic programs. On the other hand, another study [42] documented that there was no relationship between a normal BMI and the practice of healthy eating habits.

According to the current findings, most of the students were practicing moderately healthy nutrition behavior. The study also presented barriers to adherence to healthy eating. The significant limitation of the present study was the research design as it was cross-sectional, which may hinder the possibility of inferring the cause and effect relationship. The self-administered questionnaires could also be considered a bias, limiting the generalizable findings of the study.

## 5. Conclusions

It can be concluded that the eating habits of the students in this study were inadequate to some extent. The highest reported barriers were the easy availability of fast foods, high cost of healthy foods, limited time, and being too lazy to prepare a healthy diet. On the other hand, the lowest barrier was pressure from peers to engage in unhealthy eating. In relation to the association between the understanding of the concept of a healthy diet and students’ characteristics, it was found that students who were single, in the preparatory to second year academic level, and enrolled in nutrition courses had higher levels of awareness about healthy diets. A normal BMI was an indicator of healthy nutritional status. This study assessed the association between body mass index (BMI) and academic discipline and enrollment in a nutrition course. Statistically significant data indicated that the students who had a normal BMI were more aware of the concept of a healthy diet, studied medical and applied sciences, and were enrolled in nutrition courses. These findings suggest that attention should be paid to recommendations for enrolling in healthy diet and food courses as a mandatory subject in all KFU educational programs, to raise students’ awareness, practice, support, and encouragement around healthier nutrition. This could be considered part of a comprehensive set of preventive strategies. The regulations, guidelines, protocols, and policies should be derived by each college to take necessary action for improving healthy lifestyles among students. Additionally, further research should be carried out to find the best strategy to overcome barriers to adherence to a healthy diet.

## Figures and Tables

**Table 1:** Characteristics of the students (*n* = 564).

Items	*N*	%
Age (years)		
18–19	190	33.7
20–22	297	52.7
23–25	77	13.7
Mean ±SD	20.45 ± 1.64	
Gender		
Male	76	13.5
Female	488	86.5
Academic Level		
Preparatory to second year	381	67.6
Third to internship year	183	32.4
Academic Disciplines		
Medical and applied sciences	430	76.2
Humanities, arts, and social sciences	134	23.8
Marital Status		
Single	468	83.0
Married	96	17.0
Family Type		
Campus	48	8.5
Simple family	321	56.9
Joint family	195	34.6
BMI (kg/m^2^)		
Underweight (<18.4)	131	23.2
Normal (<18.5–24.9)	304	53.9
Overweight (25–29.9)	78	13.8
Obese (>30)	51	9.0
Enrollment in a Nutrition Course		
Enrolled	404	71.6
Not enrolled	160	28.4
Awareness of the Concept of a Healthy Diet		
Yes	479	84.9
No	85	15.1

*N* = Number; % = Percentage.

**Table 2:** Eating habits distribution among students (*n* = 564).

	Daily Basis				Weekly Basis				Rare		X^2^	*p* Value
How Many Times do You Eat	Once		Twice		1–2 Times		3–4 Times					
	*N*	%	*N*	%	*N*	%	*N*	%	*N*	%		
Snacks	152	27.0	258	45.7	23	4.1	38	6.7	93	16.5	325.1	0.0001 *
Vegetables	145	25.7	49	8.7	116	20.6	89	15.8	165	29.3	74.55	0.0001 *
Fruits	117	20.7	52	9.2	147	26.1	100	17.7	148	26.2	55.73	0.0001 *
Dates	108	19.1	47	8.3	76	13.5	58	10.3	275	48.8	310.45	0.0001 *
Fried food	67	11.9	21	3.7	205	36.3	117	20.7	154	27.3	83.87	0.0001 *
With family	130	23.0	216	38.3	49	8.7	109	19.3	60	10.6	157.97	0.0001 *
With friends	26	4.6	8	1.4	93	16.5	25	4.4	412	73.0	1029.6	0.0001 *
Fast food	22	3.9	10	1.8	213	37.8	80	14.2	239	42.4	406.51	0.0001 *
Balanced diet in meals	86	15.2	91	16.1	79	14.0	68	12.1	240	42.6	181.94	0.0001 *

*N* = Number; % = Percentage; X^2^ = Chi square; * statistically significant at *p* < 0.05.

**Table 3:** Frequency of dietary practices among students (*n* = 564).

Frequency Questions	Always		Sometimes		Rare		Never		X^2^	*p* Value
	*N*	%	*N*	%	*N*	%	*N*	%		
Take meals regularly	164	29.1	277	49.1	35	6.2	88	15.6	234.54	0.0001 *
Take breakfast daily	174	30.9	261	46.3	92	16.3	37	6.6	203.59	0.0001 *
Take breakfast outside home	34	6.0	219	38.8	228	40.4	83	14.7	201.89	0.0001 *
Read nutrient composition label	101	17.9	144	25.5	169	30.0	150	26.6	17.55	0.001 *
Consider plant source in main meal	93	16.5	230	40.8	128	22.7	113	20.0	79.28	0.0001 *
Consider nutrient value	121	21.5	176	31.2	140	24.8	127	22.5	12.92	0.005 *
Add iodized salt while cooking food	282	50.0	147	26.1	75	13.3	60	10.6	218.68	0.0001 *
Follow any healthy diet	54	9.6	206	36.5	112	19.9	192	34.0	108.06	0.0001 *

X^2^: Chi square; * statistically significant at *p* ≤ 0.05.

**Table 4:** Barriers to adherence to healthy eating among students (*n* = 564).

Barriers	Agree	Neutral			Disagree		X^2^	*p* Value
	*N*	%	*N*	%	*N*	%		
Student Factors								
Not interesting in eating a healthy diet	150	26.6	175	31	239	42.4	22.45	0.0001 *
No physical activity, exercise or sport	156	27.7	127	22.5	281	49.8	71.245	0.0001 *
Too lazy to prepare healthy foods	322	57.1	135	23.9	107	19.0	145.35	0.0001 *
Non-periodical follow-up of my nutritional status	271	48.0	195	34.6	98	17.4	79.98	0.0001 *
Unhealthy diet used as coping strategy for stress	207	36.7	200	35.5	157	27.8	7.798	0.020 *
Educational Factors								
Inadequate knowledge/information about healthy diets	228	40.4	162	28.7	174	30.9	13.149	0.001 *
Limited literature or books related to healthy eating	172	30.5	173	30.7	219	38.8	7.670	0.022 *
Unavailability of healthy diet	235	41.7	171	30.3	158	28.0	18.074	0.0001 *
Food Factors								
Cooking healthy food is more difficult than junk food	303	53.7	121	21.5	140	24.8	106.47	0.0001 *
Limited variety of healthy foods	295	52.3	140	24.8	129	22.9	91.67	0.0001 *
Good taste and food preferences for an unhealthy diet	286	50.7	178	31.6	100	17.7	49.04	0.0001 *
Fast foods are easily and quickly available	413	73.2	106	18.8	45	8.0	413.82	0.0001 *
Attractive advertising of unhealthy diet	313	55.5	140	24.8	111	19.7	126.90	0.0001 *
High cost of healthy foods	410	72.7	105	18.6	49	8.7	401.56	0.0001 *
Environment Factors								
College climate is not a suitable place to eat a healthy diet	268	47.5	154	27.3	142	25.2	51.447	0.0001 *
Limited time to prepare healthy foods	333	59.0	165	29.3	66	11.7	193.82	0.0001 *
Social Factors								
Family is not supportive of eating healthy diet	190	33.7	144	25.5	230	40.8	19.70	0.0001 *
Pressure from peers to engage in unhealthy eating	122	21.6	158	28.0	284	50.4	76.98	0.0001 *
Cultural and traditional food is not healthy	252	44.7	199	35.3	113	20.0	52.351	0.0001 *

X^2^: Chi square; *: statistically significant at *p* ≤ 0.05.

**Table 5:** Association between awareness of the concept of a healthy diet and student characteristics.

Students’ Characteristics	Awareness of the Healthy Diet Concept (*n* = 564)					
	Yes (*n* = 479)		No (*n* = 85)		X^2^	*p* Value
Age (Years) 18–19	*N*	%	*N*	%		
	154	32.2	36	42.4	3.921	0.141
20–22	256	53.4	41	48.2		
23–25	69	14.4	8	9.4		
Family Type						
Simple family	265	55.3	56	65.9	5.45	0.065
Campus	39	8.1	9	10.6		
Joint family	175	36.5	20	23.5		
Marital Status						
Single	408	85.2	60	70.6	10.879	0.001 *
Married	71	14.8	25	29.4		
Gender						
Male	66	13.8	10	11.8	0.251	0.616
Female	413	86.2	75	88.2		
Academic Level						
Preparatory to second year	315	65.7	66	77.6	4.65	0.031 *
Third to internship year	164	34.2	19	22.4		
Academic Disciplines						
Medical and applied sciences	305	54.1	125	22.1	0.438	0.508
Humanities, arts, and social sciences	99	17.5	35	6.2		
Nutrition Course						
Enrolled	357	74.5	47	55.3	13.34	0.0001 *
Not Enrolled	122	25.5	38	44.7		

X^2^: Chi square * statistically significant at *p* ≤ 0.05.

**Table 6:** Association between BMI and academic discipline and nutrition course attendance (*n* = 564).

Students’ BMI (kg/m2)	Awareness about Healthy Diet Concept					Academic Disciplines					Nutrition Course				
	Yes		No			Medical and Applied Sciences		Humanities and Social Sciences			Enrolled		Not Enrolled		
	*N*	%	*N*	%		*N*	%	*N*	%		*N*	%	*N*	%	
Underweight (≤18.4)	102	21.3	29	34.1	X^2^ = 7.84 *p* = 0.04 *	112	19.8	19	3.3	X^2^ = 16.452 *p =* 0.001*	89	15.7	42	7.4	X^2^ = 7.845 *p* = 0.049 *
Normal (≤18.5–24.9)	263	54.9	41	48.2		225	39.8	79	14		226	40	78	13.8	
Overweight (25–29.9)	67	14	11	12.9		63	11.1	15	2.6		48	8.5	30	5.3	
Obese (≥30)	47	9.8	4	4.7		30	5.3	21	3.7		41	7.2	10	1.7	

X^2^: Chi square * statistically significant at *p* ≤ 0.05.
